# Maternal Factors Associated with Low Birth Weight in Term Neonates: A Case-controlled Study

**DOI:** 10.1055/s-0038-1667341

**Published:** 2018-08

**Authors:** Eduardo Mahecha-Reyes, Carlos Fernando Grillo-Ardila

**Affiliations:** 1 Department of Health Sciences, Universidad Surcolombiana, Neiva, Colombia; 2 Department of Obstetrics and Gynecology, Universidad Nacional de Colombia, Bogotá, Colombia

**Keywords:** case-controlled studies, developing countries, low birth weight, risk factors, term birth, estudos de caso-controle, países em desenvolvimento, baixo peso ao nascer, fatores de risco, termo de nascimento

## Abstract

**Objective** To identify maternal factors associated with the presence of low birth weight in term neonates.

**Methods** Matched hospital-based case-controlled study performed in a high complexity institution located in the city of Neiva, Colombia. The study included women with term gestation and singleton live fetuses. Patients with prior diseases, coming from other regions, with pregnancy resulting from assisted reproduction, or with a diagnosis of fetal abnormality or aneuploidy were excluded. Low birth weight was the dependent variable, and the independent variables that were analyzed were maternal sociodemographic and clinical characteristics. Adjusted and non-adjusted odds ratios (aOR and OR) together with the 95% confidence intervals (95% CI) were reported.

**Results** The study included 270 participants (90 cases and 180 controls). Controlling for maternal age, educational level, socioeconomic and civil status, social security and the presence of maternal disease during gestation, it was found that weight gain (aOR 0.77, 95% CI 0.70–0.85) and the absence of prenatal care (aOR 8.20, 95% CI 3.22–20.87) were among the factors associated with low birth weight.

**Conclusions** The absence of weight gain and of prenatal care are factors associated with the presence of low birth weight in term neonates and should be considered in clinical practice.

## Abstract (pt)

**Resumo:**

**Objetivo** Identificar fatores maternos associados à presença de baixo peso ao nascer em neonatos a termo.

**Métodos** Estudo de caso-controle realizado em uma instituição de alta complexidade localizada na cidade de Neiva, Colômbia. O estudo incluiu mulheres com gestação a termo e fetos vivos únicos. Pacientes com doenças prévias, provenientes de outras regiões, com gravidez resultante de reprodução assistida, ou com diagnóstico de anormalidade fetal ou aneuploidia foram excluídos. O baixo peso ao nascer foi a variável dependente, e as variáveis independentes analisadas foram as características sociodemográficas e clínicas maternas. Razões de chance ajustadas e não ajustadas (RCa e RC) juntamente com os intervalos de confiança de 95% (IC 95%) foram relatadas.

**Resultados** O estudo incluiu 270 participantes (90 casos e 180 controles). Controlando a idade materna, nível escolar, socioeconômico e civil, segurança social e a presença de doença materna durante a gestação, constatou-se que ganho de peso (RCa 0,77, IC 95% 0,70–0,85) e ausência de pré-natal (RCa 8,20, IC 95% 3,22–20,87) estavam entre os fatores associados ao baixo peso ao nascer.

**Conclusão** As ausências de ganho ponderal e de pré-natal são fatores associados à presença de baixo peso ao nascer em recém-nascidos a termo e devem ser considerados na prática clínica.

## Introduction

The World Health Organization (WHO) defines low birth weight as weight at birth lower than 2,500 g.^1^ However, birth weight is determined by two critical considerations: gestational age at delivery and the rate of fetal growth.^2^ Consequently, low weight determining factors may differ between a preterm and a term neonate because, in the former, low weight is usually explained by prematurity, while in the latter, it is result of intrinsic and/or extrinsic factors that impact on developmental potential.^1^ ^2^ Therefore, it comes as no surprise that low birth weight neonates have a worse prognosis in terms of survival and neural development.^3^

According to The United Nations International Children's Emergency Fund (UNICEF), more than 20 million infants are born with low weight in the world, accounting for 15% of all births. Of these, more than 95% are born in middle- and low-income countries, with those in Asia, Africa and Latin America being the most frequently affected.^4^ Prevalence in these countries is twice as high as the ones observed in developed countries, reflecting the inequities faced by pregnant women in those regions of the world where adverse conditions, such as malnutrition, poor weight gain, anemia or pregnancy-related disorders, like hypertension, possibly explain the observed frequencies.^1^ ^5^

Preventing low birth weight is a public health priority, and one of the goals for the new millennium.^4^ Reducing the frequency of low birth weight pregnancies could have a positive impact on infant mortality in middle- and low-income countries, which are the ones with the largest shortage of resources required for the care of these neonates.^4^ Consequently, efforts aimed at identifying risk factors associated with this condition are mandatory.^6^ Knowledge of the factors that influence low birth weight will contribute to the timely identification and early intervention in pregnant women at risk.^7^ Therefore, the objective of this study was to identify maternal factors associated with the presence of low birth weight in term neonates born in 2015 and 2016 in a high complex institution in the city of Neiva, Colombia.

## Methods

This was a retrospective, analytical case-controlled study conducted at Hospital Universitario Hernando Moncaleano Perdomo, a high complexity institution located in the city of Neiva, serving the population of southwestern Colombia.

The subjects included were women with term gestations and singleton live fetuses, who were treated at the participating institution between January 2015 and October 2016. Patients with any existing diseases before pregnancy (such as hypertensive vascular disease, nephropathy, diabetes mellitus, thrombophilia, heart, neurodegenerative, cancer or autoimmune diseases), coming from a different geographic area, with a gestation resulting from assisted reproduction, or with a confirmed diagnosis of major fetal abnormality or aneuploidy were excluded.

Cases were defined as live neonates with a weight at birth under 2,500 g and a gestational age of 37 weeks of gestation or more. Gestational age was estimated based on the last menstrual period or ultrasound. Term neonates weighing 2,500 g or more were considered controls. The cases were identified using entries in the database of the epidemiological surveillance system corresponding to low birth weight, and the information was compared with the newborn vital statistics registry. In Colombia, the birth of a neonate with low weight is a public health event and reporting is mandatory. Controls were selected from the vital statistics database of the participating institution and the quality of the data was verified by means of a clinical record review. The cases and controls were selected by random sampling until the required sample size was completed.

Sample size estimation was based on the approach suggested by Freeman, consisting of the use of the event of interest by variable. In this way, the sample size for a non-conditioned logistic regression was determined to be 10 * (k + 1), in which k is the number of study variables.^8^ According to this principle, and given that for this study, *a priori,* research into the potential association between low birth weight and maternal age, weight gain, educational level, socioeconomic and marital status, social security, gestational age at the start of prenatal care, and the presence of maternal disease during gestation had been proposed, at least 90 cases and an equal number of controls were required (*n* = 180). However, it was decided to select 2 controls for every case to increase the power. Thus, the required sample size for the study was 270 participants.

The data were analyzed using the Stata software package, version 15 (StataCorp. College Station, TX, USA). Descriptive statistics were applied to clinical and sociodemographic variables. The central trend and scatter were estimated for continuous data, and proportions and frequency measurements were used for qualitative data. The frequency and distribution were examined for categorical variables, and a normal distribution and variance homogeneity were analyzed for continuous variables. For the categorical variables, differences between cases and controls were tested using the Fisher exact test or the Pearson Chi-squared test. Continuous variables were compared using the Mann-Whitney test, given the absence of a normal distribution. A univariate logistic regression was applied to assess the association between candidate variables and low birth weight.

A multivariate logistic model was built to incorporate the clinical and sociodemographic variables mentioned above. A non-conditional logistic regression was performed to adjust for the presence of potential confounding factors, the predictive ability of the model was estimated, and the goodness of the adjustment was assessed using McFadden R^2^. However, to identify the most parsimonious model, a second analysis was performed using the stepwise backward approach, to which the Bonferroni correction was applied, thus adjusting the significance level.^9^ For the second model, the independent variables associated with the outcome of interest were preserved, with a significance level lower than 0.005.^9^ Confidence intervals were estimated, and adjusted and non-adjusted odds ratios (aOR and OR) are presented as the association measure. The study protocol was approved by the Ethics Committee of the participating institution (Reference 009–006).

## Results

There were 4,882 term deliveries in the participating institution during the study period. Of this total, 3.0% were neonates with low birth weight for gestational age. Once the target population was identified, the cases and controls were selected by random sampling until the required sample size was completed. For each selected case and control, we verify the inclusion and exclusion criteria before their incorporation into study. Finally, the study population was then assembled and consisted of 90 cases and 180 controls.

In terms of the characteristics of the population analyzed, the mean age was 23 years, and there was a predominance of secondary education level, low socioeconomic bracket, free union as marital status, affiliation with the subsidized healthcare regime, and urban place of residence. Regarding clinical characteristics, 54.0% of the women were multiparous and 61.4% had attended 5 or more prenatal care visits; 62.9% were vaginal deliveries, the mean gestational age at the time of delivery was 38.4 weeks, and the mean birth weight was 2,965 g (SD ± 552 g). Table 1 summarizes the baseline characteristics of the population by group (cases or controls).

**Table 1:** Description of the sociodemographic and clinical characteristics of cases and controls

Variable Mean	Cases (*n* = 90)		Controls (*n* = 180)		*p value*
	Mean	Range	Mean	Range	
Age	22.0	(14–42)	24.1	(13–45)	*p = *0.00^a^
**Variable measured**	**n (%)**		**n (%)**		***p value***
Educational level					*p = *0.29^b^
None	0 (0.00)		1(0.56)		
Primary	20 (22.22)		30 (16.67)		
Secondary	61 (67.78)		138 (76.6)		
Technical/University	9 (10.00)		11 (6.11)		
Socioeconomic status					*p = *0.00^b^
Low	58 (64.44)		139 (77.22)		
Medium	27 (30.00)		40 (22.22)		
High	5 (5.56)		1 (0.56)		
Marital status					*p = *0.89^c^
Single	39 (43.33)		79 (43.89)		
Free union	43 (47.78)		88 (48.89)		
Married	8 (8.89)		13 (7.22)		
Social security					*p = *0.52^b^
Subsidized	83 (92.22)		154 (85.56)		
Contributive/Special	6 (6.67)		23 (12.78)		
No payment capability	1 (1.11)		3 (1.67)		
Place of residence					*p* = 0.37^c^
Urban	64 (71.11)		137 (76.11)		
Rural	26 (28.89)		43 (23.89)		
Gestational age at the start of prenatal care					*p = *0.00^c^
First trimester	30 (33.33)		86 (47.78)		
Second trimester	16 (17.78)		48 (26.67)		
Third trimester	20 (22.22)		39 (21.67)		
No prenatal care	24 (26.67)		7 (3.89)		
Disease during pregnancy					*p = *0.00^c^
No	58 (64.44)		143 (79.44)		
Yes	32 (35.56)		37 (20.56)		
Sex of the newborn					*p = *0.79^c^
Female	48 (53.33)		93 (51.67)		
Male	42 (46.67)		87 (48.33)		

a Mann-Whitney test for mean differences.

b Fisher exact test.

c Pearson Chi-squared test.

The two groups were similar, except in terms of socioeconomic status, gestational age at the start of prenatal care, and the presence of disease during pregnancy. Table 2 summarizes the frequency and type of disease by group (cases or controls). Maternal age was significantly different between the groups, although the difference observed was not clinically relevant. For the neonates (data not shown), the mean gestational age at the time of birth was 37.9 weeks for the cases and 39.0 weeks for the controls, while the average weight was 2,328 g (SD ± 166 g) for the cases and 3,282 g (SD ± 371 g) for the controls. Of the low-weight neonates, 78.2% were admitted to the kangaroo program and 3.2% to the neonatal intensive care unit.

**Table 2:** Frequency and type of disease during pregnancy by group (cases or controls)

Disease during pregnancy	Cases (n = 90)	Controls (*n* = 180)	*p*-value
	n (%)	n (%)	
No	58 (64.4)	143 (79.4)	***p*** ** = 0.00** a
Yes	32 (35.5)	37 (20.5)	
By type			
Hypertensive disorder	19 (21.1)	17 (9.4)	
Perinatal infection	6 (6.6)	7 (3.8)	
Endocrine disease	2 (2.2)	5 (2.7)	
Placental-amniotic fluid disease	4 (4.4)	2 (1.1)	
Anemia	0 (0.0)	4 (2.2)	
Neurological condition	1 (1.1)	1 (0.05)	
Pulmonary disease	0 (0.0)	1 (0.05)	

a Pearson Chi-squared test.

The univariate logistic regression revealed that maternal age (OR 0.94, 95% CI 0.90–0.98) and weight gain during pregnancy (OR 0.77, 95% CI 0.70–0.84) behaved as protective factors. The risk was also significantly lower depending on the educational level of the mother (technical/university education OR 0.10, 95% CI 0.01–0.83). On the other hand, the absence of prenatal care (OR 9.82, 95% CI 3.84–25.13) and the presence of maternal disease during pregnancy (OR 2.13, 95% CI 1.21–3.74) behaved as risk factors. Table 3 shows the results of the logistic regression, together with their respective aOR and CI.

**Table 3:** Multivariate analysis of maternal factors associated with low birth weight

Variables	First model^a^ aOR (95% CI)^b^	Second model^a^ aOR (95% CI)
Maternal age	0.91 (0.86–.96)	3^c^
Weight gain	0.77 (0.69–0.86)	0.77 (0.70–0.85)
Absence of prenatal care	10.67 (3.49–32.64)	8.20 (3.22–20.87)

Abbreviations: aOR, adjusted odds ratio; 95% CI, 95% confidence interval.

a Adjusted for variables: level of schooling, socioeconomic bracket, marital status, social security and the presence of maternal disease.

b Adjusted odds ratios together with the 95% confidence intervals;

c Variable removed from the model: *p* of 0.01 but greater than 0.005.

Following this exploration, a multivariate analysis was performed with the main goal of identifying the clinical or sociodemographic characteristics that could be linked to the presence of low birth weight. An initial model was built, ratifying the role played by maternal age, low weight gain and absence of prenatal care. When the goodness of the adjustment was assessed, the McFadden R^2^ was 0.27, with a predictive capacity of 80.3%, reflecting a high percentage of correctness.^10^ However, when the second analysis was performed, only the absence of prenatal care and of adequate weight gain continued to be statistically significant. This time, the McFadden R^2^ was 0.17, with a predictive capacity of 72.4%, showing an acceptable percentage of correctness.^10^

## Discussion

Low birth weight is one of the most important determinants of infant morbidity and mortality because it increases the frequency of adverse perinatal outcomes.^11^ Identification of factors that may have an impact on potential fetal growth is an excellent opportunity to have an impact on the health conditions of the population.^12^

There were 4,882 term live births during the observation period, of which 3.0% were low birth weight neonates. The population consisted mainly of young, single mothers with low socioeconomic and education levels, living in urban areas. Only 43% of the women started prenatal care during the first trimester of gestation. In our study, the prevalence of low birth weight was similar to that reported in other Latin American countries, but substantially lower than the one described for countries in Southeast Asia.^13^ ^14^ ^15^ ^16^ ^17^ ^18^ ^19^ These differences could be explained, at least in part, by the rate of fetal growth, genetic factors and the presence of other extrinsic circumstances not related to pregnancy.^7^ ^20^

On the other hand, regarding the multivariate analysis, it revealed that maternal weight gain (aOR 0.77, 95% CI 0.70–0.85) behaves as a protective factor, while the absence of prenatal care (aOR 8.20, 95% CI 3.22–20.87) increases the probability of an unfavorable outcome. Our findings are similar to those documented in the literature. Observational studies have shown the association between maternal weight gain and neonatal birth weight.^7^ For example, it is known that infants born to mothers with poor weight gain are at a higher risk of being small for gestational age, while those born to mothers with substantial weight gain have a higher probability of being large.^21^ This association is consistent in low, middle and high-income countries alike.^22^

Regarding poor prenatal care or absence thereof, the observed association emerges in populations from middle and low-income countries, and it is not completely clear in developed countries.^7^ ^23^ ^24^ ^25^ ^26^ ^27^ The potential explanation is that early and adequate prenatal care could be of greater benefit in women with less favorable conditions, where the implementation of this intervention could help address harmful behaviors that affect fetal growth (for example, smoking), early detection and treatment of diseases affecting gestation (such as anemia, malnutrition), and promote healthy lifestyle habits that can have a positive impact on the fetal environment.^20^ ^28^

Finally, although maternal age was eliminated as a variable in the second model, the observed association seems plausible, is clinically relevant, and has been documented in other studies.^29^ This association could be explained by the conditions of inequity and disadvantage faced by teen mothers when compared with older women. Therefore, not surprisingly, the risk of low birth weight decreases as a function of older maternal age, given that older women may have better socioeconomic conditions and find themselves less at a social disadvantage.^30^ It is no secret that racial segregation and deprivation are associated with low birth weight.^31^ Notwithstanding, it needs to be said that this conclusion must be interpreted cautiously, given that the finding could be the result of multiple comparisons.

This study has some strengths, starting with the appropriate and widely accepted definition of the cases, which are considered representative of the study population because all the eligible neonates with the outcome of interest were included. In Colombia, reporting of low birth weight events is mandatory, hence there is a low probability of having missed a case.^32^ ^33^ On the other hand, given matched and independent data recording, there is confidence regarding the reliability of the data and a low probability of error in the definition of cases and controls.

Case-controlled comparability was achieved by means of the design and the analysis.^34^ In the design, stringent inclusion and exclusion criteria were used in an attempt to arrive at a relatively homogenous population. In the analysis, a mathematical model was used to adjust for the presence of confounding factors. Although it is true that excluding the presence of residual confounding factors is not feasible, given the nature of the design, the importance and number of the variables considered allows, in some way, for acceptable case-controlled comparability.^35^ Finally, the other strength of this study is that exposure was proven through entry in the clinical record and in the vital statistics database of the Health Secretariat, reducing the risk of poor classification.^35^

This study also has weaknesses. The subjects used as controls came from the same institution and not from the community, making the study prone to selection bias (Berkson fallacy) because, given the origin of the controls, they could be more prone to having certain factors associated with low birth weight, leading to potential distortions for some exposure-disease associations.^35^ Sample size is yet another weakness of the study. Although a design was developed *a priori* for estimating sample size, the critical assessment of the confidence intervals points to some degree of inaccuracy.^36^

Despite its limitations, this study has many practical implications. First, it highlights the need to ensure adequate weight gain during pregnancy. This is important because adequate weight gain during gestation behaves as an indirect indicator of good nutritional status of the pregnant woman. Secondly, the association observed between poor prenatal care and low birth weight should prompt timely and adequate access to medical care during gestation, especially for vulnerable populations. Medical care during the reproductive period is a valuable opportunity to have a positive impact on health conditions by means of education regarding healthy lifestyle and to ensure early detection and treatment of diseases affecting gestation. Finally, the role of an unfavorable socioeconomic environment could play out in the association observed between maternal age and low birth weight. Hence the need for highlighting the relevance of providing timely and equitable access to quality healthcare systems.

## Conclusion

Based on the findings of this study, low maternal weight gain and untimely initiation of prenatal care are some of the factors known to be associated with the presence of low birth weight in term neonates. On the other hand, the role of maternal age could also be relevant, considering that this association reflects, at least in part, the conditions of poverty and deprivation of the study population. This study, despite its many strengths, has limitations. Therefore, further studies are required to undertake a more extensive evaluation of the maternal factors associated with the presence of low birth weight among term neonates.
